# Supplementary material for: Probing metazoan polyphosphate biology using Drosophila reveals novel and conserved polyP functions
Source: eLife. 2026 Apr 8;14:RP104841. doi: 10.7554/eLife.104841 (PMC13061417; doi:10.7554/eLife.104841)
Supplement: Supplementary file 1. [file elife-104841-supp1.docx]

**Supporting Information**

**Fly lines:**

| **Name** | **Flybase ID** | **References** |
| --- | --- | --- |
| *Canton-S* | FBsn0000274 |  |
| *Tub-GAL4* | FBtp0002651 |  |
| *Cg-GAL4* | FBtp0012452 | [(3)](https://sciwheel.com/work/citation?ids=739193&pre=&suf=&sa=0) |
| *Hml-GAL4 > UAS-GFP* | FBst0030140 | [(4)](https://sciwheel.com/work/citation?ids=14584035&pre=&suf=&sa=0) |
| *Lz-GAL4 > UAS-mCD8-GFP* | FBst0006314 | [(5)](https://sciwheel.com/work/citation?ids=14049197&pre=&suf=&sa=0) |
| *NosGAL4 VP16* | FBti0131634 | [(6)](https://sciwheel.com/work/citation?ids=318565&pre=&suf=&sa=0) |
| *y ; AttP40* | FBti0114379 | [(7)](https://sciwheel.com/work/citation?ids=29514&pre=&suf=&sa=0) |
| Cyto-FLYX: *w[*] ; p(pUAST-HA-ScPpx1)AttP40 / Cyo* | This work |  |
| Nuc-FLYX: *w[*] ; p(pUAST-3X_Nuc-HA-ScPpx1)AttP40 / CyO* | This work |  |
| ER-FLYX: *w[*] ;; p(pUAST-ER-HA-ScPpx1)AttP2 / TM3,Sb* | This work |  |
| Mito-FLYX: *w[*] ;; p(pUAST-Mito-HA-ScPpx1)AttP2 / TM3,Sb* | This work |  |
| pCyto-FLYX: *w[*] ; p(pUASp-HA-ScPpx1) / CyO* | This work |  |

**Chemicals:**

| **#** | **Name** | **Company** | **Catalogue ID** |
| --- | --- | --- | --- |
| 1 | 100% molecular grade ethanol (diluent for DNA extraction) | Himedia | MB228 |
| 2 | 4% paraformaldehyde | Himedia | TCL119 |
| 3 | Acid phenol | Himedia | MB081 |
| 4 | Ammonium Molybdate | Himedia | GRM307 |
| 5 | BL21 DE3 competent cells | Himedia | MBT143 |
| 6 | Boric acid | Himedia | MB007 |
| 7 | Bovine Serum Albumin | Himedia | MB083 |
| 8 | Cell Mask Deep Red | Invitrogen | C10046 |
| 9 | Chloroform | Himedia | MB019 |
| 10 | DAPI hydrochloride | Himedia | MB097 |
| 11 | DL-DTT | Himedia | MB070 |
| 12 | EDTA | Himedia | GRM1370 |
| 13 | Goat anti-mouse-alexa555 | Invitrogen | A32727 |
| 14 | Goat anti-rabbit-alexa-488 | Invitrogen | A11034 |
| 15 | HEPES | Himedia | MB016 |
| 16 | HPLC grade water | Himedia | AS077 |
| 17 | Imidazole | Himedia | MB019 |
| 18 | L glutathione reduced | Sigma | G4251 |
| 19 | Lauryl sulphate (SDS) | Himedia | MB010 |
| 20 | Lithium chloride | Himedia | MB038 |
| 21 | Luria Broth | Himedia | M575 |
| 22 | Luria Broth Agar | Himedia | M1151 |
| 23 | Malachite Green Carbinol Hydrochloride | Glentham Life Sciences | GD8088 |
| 24 | Mouse anti-HA | Cell Signaling Technology | 2367 |
| 25 | Mouse anti-GST | Cell Signaling Technology | 2624 |
| 26 | Mouse anti-KDEL | Abcam | ab12223 |
| 27 | Mouse anti-ATP5A | Abcam | ab14748 |
| 28 | Ni-NTA agarose | Qiagen | 30210 |
| 29 | PolyP_14_, PolyP_65_, PolyP_130_ (Ex-polyP set) | Regenetiss | Gifted by T.Shiba |
| 30 | Pierce glutathione agarose | Thermo scientific | 16100 |
| 31 | Potassium phosphate dibasic anhydrous | Himedia | MB044 |
| 32 | Rabbit Anti-Fibrillarin | CST | 2639 |
| 33 | Rabbit Anti-HA | CST | 3724S |
| 34 | RNase A | Himedia | DS0003 |
| 35 | Sodium chloride | Himedia | MB023 |
| 36 | Sodium hydroxide | Himedia | MB095 |
| 37 | Tris free base | Himedia | MB029 |
| 38 | Triton X100 | Himedia | MB031 |

| **Serial number** | **Plasmid name** | **Source** |
| --- | --- | --- |
| 1 | pTrc-HisB-*ScPpx1* | Gifted by Adolfo Saiardi (*ScPpx1* gene ID- SGD:S000001244) |
| 2 | pGEX-6P2-GST-PPBD | This work |
| 3 | pGEX-6P2-GST-PPBD8Mut | This work |
| 4 | pGEX-6P2-GST | This work |

**qRT-PCR primers**

| **Gene** | **Forward Primer (5’-3’)** | **Reverse Primer (5’-3’)** | **References** |
| --- | --- | --- | --- |
| *ecd* | CTGGCGGAGTTCTTAGATCG | GCATGGAGGGATTCTTCTTG | [(8)](https://sciwheel.com/work/citation?ids=7244567&pre=&suf=&sa=0) |
| *ImPL2* | GCCGATACCTTCGTGTATCC | TTTCCGTCGTCAATCCAATAG | [(9)](https://sciwheel.com/work/citation?ids=13423123&pre=&suf=&sa=0) |
| *Eip74EF* | TCCACAATCTGCTTAGCGGC | GACTGGGCGGAAATGAACCT | [(10)](https://sciwheel.com/work/citation?ids=7279800&pre=&suf=&sa=0) |
| *inR* | AACAGTGGCGGATTCGGTT | TACTCGGAGCATTGGAGGCAT | [(11)](https://sciwheel.com/work/citation?ids=17012083&pre=&suf=&sa=0) |
| *chico* | GGCATACGGGCAGCTAGAC | TTCTTGAGGTAGCCACTCAGC | [(11)](https://sciwheel.com/work/citation?ids=17012083&pre=&suf=&sa=0) |
| *dfoxo* | TCGAGTGCAATGTCGAGGAG | AGCGGTATATTGATGTCCAGCAG | [(11)](https://sciwheel.com/work/citation?ids=17012083&pre=&suf=&sa=0) |
| *dilp2* | ATGGTGTGCGAGGAGTATAATCC | TCGGCACCGGGCATG | [(12)](https://sciwheel.com/work/citation?ids=5359496&pre=&suf=&sa=0) |
| *s6k* | TGACCTAGAACCGGAATTGTG | TCCTCGCAGAGCTGTATGG | [(11)](https://sciwheel.com/work/citation?ids=17012083&pre=&suf=&sa=0) |
| *4ebp* | CACTCCTGGAGGCACCA | GAGTTCCCCTCAGCAAGCAA | [(12)](https://sciwheel.com/work/citation?ids=5359496&pre=&suf=&sa=0) |
| *tor* | GCTCAGAGGCGAGAGACAAG | CCAGCTCACGGAGGATAAAG | [(11)](https://sciwheel.com/work/citation?ids=17012083&pre=&suf=&sa=0) |
| *rp49* | TCCTACCAGCTTCAAGATGAC | CACGTTGTGCACCAGGAACT | This work |

**Sequence of codon optimised *HA*-*ScPpx1* for creation of Cyto-FLYX system**

ATGTATCCGTATGATGTTCCGGATTATGCATCGCCTTTGAGAAAGACGGTTCCTGAATTTTTGGCACACTTAAAATCACTGCCAATTTCAAAAATTGCAAGCAACGATGTGTTAACAATATGTGTTGGTAACGAGTCAGCAGATATGGACTCAATTGCTAGTGCAATCACTTATTCGTACTGCCAATACATATATAATGAAGGTACTTACTCGGAGGAGAAAAAGAAAGGAAGCTTTATTGTCCCAATTATCGACATTCCTAGAGAAGACCTCAGTTTAAGAAGAGACGTAATGTATGTCCTAGAAAAACTGAAAATTAAGGAGGAGGAATTATTCTTCATTGAAGATTTAAAGAGCTTGAAGCAAAATGTCTCCCAGGGTACTGAATTAAACTCTTACTTGGTAGATAATAACGATACACCAAAGAATTTGAAAAATTATATAGATAACGTCGTTGGCATTATAGACCACCATTTTGACTTGCAAAAACATTTGGATGCTGAACCTCGGATTGTAAAAGTGTCCGGCAGTTGCTCATCGCTGGTTTTTAACTACTGGTATGAAAAATTGCAAGGTGACCGTGAAGTGGTGATGAACATTGCACCACTTTTGATGGGGGCCATCTTAATAGACACTTCAAATATGAGGCGCAAAGTCGAGGAAAGTGATAAATTAGCTATCGAGAGATGCCAAGCTGTTCTTAGTGGTGCGGTTAATGAAGTGTCTGCGCAAGGTTTAGAGGACAGCAGTGAGTTTTATAAAGAGATAAAATCAAGAAAGAACGATATTAAAGGATTTTCGGTAAGCGATATTCTAAAGAAGGACTACAAACAATTCAATTTCCAAGGAAAGGGACACAAAGGGTTAGAGATTGGTCTTTCATCAATAGTAAAAAGAATGTCTTGGCTATTCAATGAACACGGTGGTGAAGCAGATTTCGTCAACCAATGCAGAAGATTTCAGGCGGAGAGGGGGCTCGATGTATTGGTTCTGTTGACTTCATGGAGGAAAGCTGGTGATTCACACAGAGAATTGGTCATATTGGGAGACTCTAACGTGGTACGTGAACTCATTGAAAGGGTTAGCGACAAGCTCCAACTTCAATTATTTGGGGGCAATCTTGATGGAGGTGTGGCGATGTTTAAGCAACTGAACGTCGAGGCCACCAGAAAGCAAGTCGTCCCCTATTTAGAGGAAGCGTACTCAAACCTGGAAGAGTGA

**Sequence of codon optimised 3X-NLS-*HA*-*ScPpx1* for creation of Nuc-FLYX system**

ATGCCCAAGAAGAAGAGGAAGGTTCCCAAGAAGAAGAGGAAGGTTCCCAAGAAGAAGAGGAAGGTTGGCGGCCGCTATCCGTATGATGTTCCGGATTATGCATCGCCTTTGAGAAAGACGGTTCCTGAATTTTTGGCACACTTAAAATCACTGCCAATTTCAAAAATTGCAAGCAACGATGTGTTAACAATATGTGTTGGTAACGAGTCAGCAGATATGGACTCAATTGCTAGTGCAATCACTTATTCGTACTGCCAATACATATATAATGAAGGTACTTACTCGGAGGAGAAAAAGAAAGGAAGCTTTATTGTCCCAATTATCGACATTCCTAGAGAAGACCTCAGTTTAAGAAGAGACGTAATGTATGTCCTAGAAAAACTGAAAATTAAGGAGGAGGAATTATTCTTCATTGAAGATTTAAAGAGCTTGAAGCAAAATGTCTCCCAGGGTACTGAATTAAACTCTTACTTGGTAGATAATAACGATACACCAAAGAATTTGAAAAATTATATAGATAACGTCGTTGGCATTATAGACCACCATTTTGACTTGCAAAAACATTTGGATGCTGAACCTCGGATTGTAAAAGTGTCCGGCAGTTGCTCATCGCTGGTTTTTAACTACTGGTATGAAAAATTGCAAGGTGACCGTGAAGTGGTGATGAACATTGCACCACTTTTGATGGGGGCCATCTTAATAGACACTTCAAATATGAGGCGCAAAGTCGAGGAAAGTGATAAATTAGCTATCGAGAGATGCCAAGCTGTTCTTAGTGGTGCGGTTAATGAAGTGTCTGCGCAAGGTTTAGAGGACAGCAGTGAGTTTTATAAAGAGATAAAATCAAGAAAGAACGATATTAAAGGATTTTCGGTAAGCGATATTCTAAAGAAGGACTACAAACAATTCAATTTCCAAGGAAAGGGACACAAAGGGTTAGAGATTGGTCTTTCATCAATAGTAAAAAGAATGTCTTGGCTATTCAATGAACACGGTGGTGAAGCAGATTTCGTCAACCAATGCAGAAGATTTCAGGCGGAGAGGGGGCTCGATGTATTGGTTCTGTTGACTTCATGGAGGAAAGCTGGTGATTCACACAGAGAATTGGTCATATTGGGAGACTCTAACGTGGTACGTGAACTCATTGAAAGGGTTAGCGACAAGCTCCAACTTCAATTATTTGGGGGCAATCTTGATGGAGGTGTGGCGATGTTTAAGCAACTGAACGTCGAGGCCACCAGAAAGCAAGTCGTCCCCTATTTAGAGGAAGCGTACTCAAACCTGGAAGAGTGA

**Sequence of codon optimised Mito-*HA*-*ScPpx1* for creation of Mito-FLYX system**

ATGTCCGTCCTGACGCCGCTGCTGCTGCGGGGCTTGACAGGCTCGGCCCGGCGGCTCCCAGTGCCGCGCGCCAAGATCCATTCGTTTGCCGCCGGCGGCCGCTATCCGTATGATGTTCCGGATTATGCATCGCCTTTGAGAAAGACGGTTCCTGAATTTTTGGCACACTTAAAATCACTGCCAATTTCAAAAATTGCAAGCAACGATGTGTTAACAATATGTGTTGGTAACGAGTCAGCAGATATGGACTCAATTGCTAGTGCAATCACTTATTCGTACTGCCAATACATATATAATGAAGGTACTTACTCGGAGGAGAAAAAGAAAGGAAGCTTTATTGTCCCAATTATCGACATTCCTAGAGAAGACCTCAGTTTAAGAAGAGACGTAATGTATGTCCTAGAAAAACTGAAAATTAAGGAGGAGGAATTATTCTTCATTGAAGATTTAAAGAGCTTGAAGCAAAATGTCTCCCAGGGTACTGAATTAAACTCTTACTTGGTAGATAATAACGATACACCAAAGAATTTGAAAAATTATATAGATAACGTCGTTGGCATTATAGACCACCATTTTGACTTGCAAAAACATTTGGATGCTGAACCTCGGATTGTAAAAGTGTCCGGCAGTTGCTCATCGCTGGTTTTTAACTACTGGTATGAAAAATTGCAAGGTGACCGTGAAGTGGTGATGAACATTGCACCACTTTTGATGGGGGCCATCTTAATAGACACTTCAAATATGAGGCGCAAAGTCGAGGAAAGTGATAAATTAGCTATCGAGAGATGCCAAGCTGTTCTTAGTGGTGCGGTTAATGAAGTGTCTGCGCAAGGTTTAGAGGACAGCAGTGAGTTTTATAAAGAGATAAAATCAAGAAAGAACGATATTAAAGGATTTTCGGTAAGCGATATTCTAAAGAAGGACTACAAACAATTCAATTTCCAAGGAAAGGGACACAAAGGGTTAGAGATTGGTCTTTCATCAATAGTAAAAAGAATGTCTTGGCTATTCAATGAACACGGTGGTGAAGCAGATTTCGTCAACCAATGCAGAAGATTTCAGGCGGAGAGGGGGCTCGATGTATTGGTTCTGTTGACTTCATGGAGGAAAGCTGGTGATTCACACAGAGAATTGGTCATATTGGGAGACTCTAACGTGGTACGTGAACTCATTGAAAGGGTTAGCGACAAGCTCCAACTTCAATTATTTGGGGGCAATCTTGATGGAGGTGTGGCGATGTTTAAGCAACTGAACGTCGAGGCCACCAGAAAGCAAGTCGTCCCCTATTTAGAGGAAGCGTACTCAAACCTGGAAGAGTGA

**Sequence of codon optimised ER-*HA*-*ScPpx1* for creation of ER-FLYX system**

ATGAGCTTTGTGAGCCTGCTGCTGGTGGGCATTCTGTTTTGGGCGACCGAAGCGGAACAGCTGACCAAATGCGAAGTGTTTTGCGGCCGCTATCCGTATGATGTTCCGGATTATGCATCGCCTTTGAGAAAGACGGTTCCTGAATTTTTGGCACACTTAAAATCACTGCCAATTTCAAAAATTGCAAGCAACGATGTGTTAACAATATGTGTTGGTAACGAGTCAGCAGATATGGACTCAATTGCTAGTGCAATCACTTATTCGTACTGCCAATACATATATAATGAAGGTACTTACTCGGAGGAGAAAAAGAAAGGAAGCTTTATTGTCCCAATTATCGACATTCCTAGAGAAGACCTCAGTTTAAGAAGAGACGTAATGTATGTCCTAGAAAAACTGAAAATTAAGGAGGAGGAATTATTCTTCATTGAAGATTTAAAGAGCTTGAAGCAAAATGTCTCCCAGGGTACTGAATTAAACTCTTACTTGGTAGATAATAACGATACACCAAAGAATTTGAAAAATTATATAGATAACGTCGTTGGCATTATAGACCACCATTTTGACTTGCAAAAACATTTGGATGCTGAACCTCGGATTGTAAAAGTGTCCGGCAGTTGCTCATCGCTGGTTTTTAACTACTGGTATGAAAAATTGCAAGGTGACCGTGAAGTGGTGATGAACATTGCACCACTTTTGATGGGGGCCATCTTAATAGACACTTCAAATATGAGGCGCAAAGTCGAGGAAAGTGATAAATTAGCTATCGAGAGATGCCAAGCTGTTCTTAGTGGTGCGGTTAATGAAGTGTCTGCGCAAGGTTTAGAGGACAGCAGTGAGTTTTATAAAGAGATAAAATCAAGAAAGAACGATATTAAAGGATTTTCGGTAAGCGATATTCTAAAGAAGGACTACAAACAATTCAATTTCCAAGGAAAGGGACACAAAGGGTTAGAGATTGGTCTTTCATCAATAGTAAAAAGAATGTCTTGGCTATTCAATGAACACGGTGGTGAAGCAGATTTCGTCAACCAATGCAGAAGATTTCAGGCGGAGAGGGGGCTCGATGTATTGGTTCTGTTGACTTCATGGAGGAAAGCTGGTGATTCACACAGAGAATTGGTCATATTGGGAGACTCTAACGTGGTACGTGAACTCATTGAAAGGGTTAGCGACAAGCTCCAACTTCAATTATTTGGGGGCAATCTTGATGGAGGTGTGGCGATGTTTAAGCAACTGAACGTCGAGGCCACCAGAAAGCAAGTCGTCCCCTATTTAGAGGAAGCGTACTCAAACCTGGAAGAGTGA

**Sequence of GST-PPBD**

ATGTCCCCTATACTAGGTTATTGGAAAATTAAGGGCCTTGTGCAACCCACTCGACTTCTTTTGGAATATCTTGAAGAAAAATATGAAGAGCATTTGTATGAGCGCGATGAAGGTGATAAATGGCGAAACAAAAAGTTTGAATTGGGTTTGGAGTTTCCCAATCTTCCTTATTATATTGATGGTGATGTTAAATTAACACAGTCTATGGCCATCATACGTTATATAGCTGACAAGCACAACATGTTGGGTGGTTGTCCAAAAGAGCGTGCAGAGATTTCAATGCTTGAAGGAGCGGTTTTGGATATTAGATACGGTGTTTCGAGAATTGCATATAGTAAAGACTTTGAAACTCTCAAAGTTGATTTTCTTAGCAAGCTACCTGAAATGCTGAAAATGTTCGAAGATCGTTTATGTCATAAAACATATTTAAATGGTGATCATGTAACCCATCCTGACTTCATGTTGTATGACGCTCTTGATGTTGTTTTATACATGGACCCAATGTGCCTGGATGCGTTCCCAAAATTAGTTTGTTTTAAAAAACGTATTGAAGCTATCCCACAAATTGATAAGTACTTGAAATCCAGCAAGTATATAGCATGGCCTTTGCAGGGCTGGCAAGCCACGTTTGGTGGTGGCGACCATCCTCCAAAATCGGATCTGGAAGTTCTGTTCCAGGGGCCCCTGGGATCCATGGATCTGTATGATGATGATGATAAAGAATTCATGGAAGGACGTTTCCGTCATCAGGATGTGCGTAGTCGCACCGCCAGCAGCCTCGCCAACCAGTATCACATCGACAGCGAACAGGCCCGACGAGTGCTGGATACCACTATGCAAATGTACGAACAGTGGCGGGAACAGCAACCGAAGCTGGCGCATCCGCAACTGGAGGCGCTACTGCGATGGGCCGCCATGCTGCATGAGGTCGGGTTGAATATCAACCACAGCGGTTTGCATCGCCACTCCGCTTATATTCTGCAAAACAGTGACTTGCCGGGTTTTAATCAGGAACAGCAGCTGATGATGGCGACACTGGTGCGCTATCACCGTAAAGCGATTAAGCTCGACGATCTGCCGCGCTTTACCTTGTTTAAGAAGAAACAGTTCCTGCCACTGATACAGCTATTGCGCCTTGGCGTATTACTAAACAATCAACGTCAGGCAACCACCACACCGCCAACATTGACACTGATTACTGATGACAGTCACTGGACACTGCGTTTCCCGCATGACTGGTTTAGTCAGAATGCGCTGGTACTGCTTGATCTGGAAAAGGAGCAAGAATACTGGGAAGGCGTGGCTGGCTGGCGGTTGAAAATTGAAGAAGAAAGTACACCTGAAATCGCCGCTTAA

**Protein sequence with Histidines, Arginines, and Lysines that are predicted for polyP binding are underlined**

MSPILGYWKIKGLVQPTRLLLEYLEEKYEEHLYERDEGDKWRNKKFELGLEFPNLPYYIDGDVKLTQSMAIIRYIADKHNMLGGCPKERAEISMLEGAVLDIRYGVSRIAYSKDFETLKVDFLSKLPEMLKMFEDRLCHKTYLNGDHVTHPDFMLYDALDVVLYMDPMCLDAFPKLVCFKKRIEAIPQIDKYLKSSKYIAWPLQGWQATFGGGDHPPKSDLEVLFQGPLGSMDLYDDDDKEFMEGRFRHQDVRSRTASSLANQYHIDSEQARRVLDTTMQMYEQWREQQPKLAHPQLEALLRWAAMLHEVGLNINHSGLHRHSAYILQNSDLPGFNQEQQLMMATLVRYHRKAIKLDDLPRFTLFKKKQFLPLIQLLRLGVLLNNQRQATTTPPTLTLITDDSHWTLRFPHDWFSQNALVLLDLEKEQEYWEGVAGWRLKIEEESTPEIAA*

**Sequence of GST-PPBD^Mut^**

ATGTCCCCTATACTAGGTTATTGGAAAATTAAGGGCCTTGTGCAACCCACTCGACTTCTTTTGGAATATCTTGAAGAAAAATATGAAGAGCATTTGTATGAGCGCGATGAAGGTGATAAATGGCGAAACAAAAAGTTTGAATTGGGTTTGGAGTTTCCCAATCTTCCTTATTATATTGATGGTGATGTTAAATTAACACAGTCTATGGCCATCATACGTTATATAGCTGACAAGCACAACATGTTGGGTGGTTGTCCAAAAGAGCGTGCAGAGATTTCAATGCTTGAAGGAGCGGTTTTGGATATTAGATACGGTGTTTCGAGAATTGCATATAGTAAAGACTTTGAAACTCTCAAAGTTGATTTTCTTAGCAAGCTACCTGAAATGCTGAAAATGTTCGAAGATCGTTTATGTCATAAAACATATTTAAATGGTGATCATGTAACCCATCCTGACTTCATGTTGTATGACGCTCTTGATGTTGTTTTATACATGGACCCAATGTGCCTGGATGCGTTCCCAAAATTAGTTTGTTTTAAAAAACGTATTGAAGCTATCCCACAAATTGATAAGTACTTGAAATCCAGCAAGTATATAGCATGGCCTTTGCAGGGCTGGCAAGCCACGTTTGGTGGTGGCGACCATCCTCCAAAATCGGATCTGGAAGTTCTGTTCCAGGGGCCCCTGGGATCCATGGATCTGTATGATGATGATGATAAAGAATTCATGGAAGGACGGTTCCGCCATCAGGATGTGCGATCACGCACAGCGTCTTCGCTCGCCAACCAGTACCACATTGATTCCGAACAAGCCCGCCGCGTTTTGGACACGACTATGCAAATGTACGAACAGTGGCGGGAGCAGCAGCCCAAGTTGGCTCACCCTCAGCTAGAAGCACTTCTAAGGTGGGCGGCCATGTTGCACGAGGTAGGCCTCAATATTAATGCAAGTGGTCTGGCTGCAGCCTCGGCATATATCCTTCAAAACAGCGACCTGCCGGGATTTAATCAGGAGCAACAGCTCATGATGGCTACGCTAGTGCGCTACGCCGCCGCCGCCATTGCGCTGGACGACCTCCCACGTTTCACCTTGTTCAAGAAAAAGCAGTTTCTGCCACTGATACAATTACTGAGACTGGGGGTGCTCTTGAATAACCAGCGTCAGGCCACTACCACGCCGCCCACATTGACACTGATCACCGATGATTCCCATTGGACCCTGCGATTTCCCCACGATTGGTTCAGCCAGAACGCCCTGGTCCTTCTGGACCTGGAGAAGGAACAGGAGTATTGGGAGGGCGTCGCGGGCTGGAGGCTGAAAATCGAGGAGGAGAGCACCCCGGAGATCGCGGCTTAA

**Protein sequence with Alanines mutation (underlined):**

MSPILGYWKIKGLVQPTRLLLEYLEEKYEEHLYERDEGDKWRNKKFELGLEFPNLPYYIDGDVKLTQSMAIIRYIADKHNMLGGCPKERAEISMLEGAVLDIRYGVSRIAYSKDFETLKVDFLSKLPEMLKMFEDRLCHKTYLNGDHVTHPDFMLYDALDVVLYMDPMCLDAFPKLVCFKKRIEAIPQIDKYLKSSKYIAWPLQGWQATFGGGDHPPKSDLEVLFQGPLGSMDLYDDDDKEFMEGRFRHQDVRSRTASSLANQYHIDSEQARRVLDTTMQMYEQWREQQPKLAHPQLEALLRWAAMLHEVGLNINASGLAAASAYILQNSDLPGFNQEQQLMMATLVRYAAAAIALDDLPRFTLFKKKQFLPLIQLLRLGVLLNNQRQATTTPPTLTLITDDSHWTLRFPHDWFSQNALVLLDLEKEQEYWEGVAGWRLKIEEESTPEIAA*

**Code for RNA Seq Analysis:**

**Step01: Import and Normalization**

# Libraries required----

library(tidyverse)

library(tximport)

library(ensembldb)

library(biomaRt)

library(edgeR)

library(matrixStats)

library(cowplot)

# Import of kallisto counting

targets <- read_tsv("studydesign.txt")

path <- file.path(targets$sample, "abundance.tsv")

myMart <- useMart(biomart="ENSEMBL_MART_ENSEMBL")

available.datasets <- listDatasets(myMart)

drosophila.anno <- useMart(biomart = "ENSEMBL_MART_ENSEMBL",

dataset = "dmelanogaster_gene_ensembl")

drosophila.attributes <- listAttributes(drosophila.anno)

Tx.drosophila <- getBM(attributes = c('ensembl_transcript_id',

'external_gene_name'),

mart = drosophila.anno)

Tx.drosophila<- as.tibble(Tx.drosophila)

Tx.drosophila <- dplyr::rename(Tx.drosophila, target_id = ensembl_transcript_id,

gene_name = external_gene_name)

Tx.drosophila <- dplyr::select(Tx.drosophila, "target_id",

"gene_name")

Txi_gene <- tximport(path,

type = "kallisto",

tx2gene = Tx.drosophila,

txOut = FALSE,

countsFromAbundance = "lengthScaledTPM",

ignoreTxVersion = TRUE)

# Data Wrangling----

myCounts <- Txi_gene$counts

sampleLabels <- targets$sample

myDGEList <- DGEList(myCounts)

cpm <- cpm(myDGEList)

log2.cpm <- cpm(myDGEList, log=TRUE)

log2.cpm.df <- as_tibble(log2.cpm, rownames = "geneID")

colnames(log2.cpm.df) <- c("geneID", sampleLabels)

log2.cpm.df.pivot <- pivot_longer(log2.cpm.df,

cols = A2:P4,

names_to = "samples",

values_to = "expression")

p1 <- ggplot(log2.cpm.df.pivot) +

aes(x=samples, y=expression, fill=samples) +

geom_violin(trim = FALSE, show.legend = FALSE) +

stat_summary(fun = "median",

geom = "point",

shape = 95,

size = 10,

color = "black",

show.legend = FALSE) +

labs(y="log2 expression", x = "sample",

title="Log2 Counts per Million (CPM)",

subtitle="unfiltered, non-normalized",

caption=paste0("produced on ", Sys.time())) +

theme_bw()

keepers <- rowSums(cpm>1)>=2

myDGEList.filtered <- myDGEList[keepers,]

log2.cpm.filtered <- cpm(myDGEList.filtered, log=TRUE)

log2.cpm.filtered.df <- as_tibble(log2.cpm.filtered, rownames = "geneID")

colnames(log2.cpm.filtered.df) <- c("geneID", sampleLabels)

log2.cpm.filtered.df.pivot <- pivot_longer(log2.cpm.filtered.df,

cols = A2:P4,

names_to = "samples",

values_to = "expression")

p2 <- ggplot(log2.cpm.filtered.df.pivot) +

aes(x=samples, y=expression, fill=samples) +

geom_violin(trim = FALSE, show.legend = FALSE) +

stat_summary(fun = "median",

geom = "point",

shape = 95,

size = 10,

color = "black",

show.legend = FALSE) +

labs(y="log2 expression", x = "sample",

title="Log2 Counts per Million (CPM)",

subtitle="filtered, non-normalized",

caption=paste0("produced on ", Sys.time())) +

theme_bw()

myDGEList.filtered.norm <- calcNormFactors(myDGEList.filtered, method = "TMM")

log2.cpm.filtered.norm <- cpm(myDGEList.filtered.norm, log=TRUE)

log2.cpm.filtered.norm.df <- as_tibble(log2.cpm.filtered.norm, rownames = "geneID")

colnames(log2.cpm.filtered.norm.df) <- c("geneID", sampleLabels)

log2.cpm.filtered.norm.df.pivot <- pivot_longer(log2.cpm.filtered.norm.df,

cols = A2:P4,

names_to = "samples",

values_to = "expression")

p3 <- ggplot(log2.cpm.filtered.norm.df.pivot) +

aes(x=samples, y=expression, fill=samples) +

geom_violin(trim = FALSE, show.legend = FALSE) +

stat_summary(fun = "median",

geom = "point",

shape = 95,

size = 10,

color = "black",

show.legend = FALSE) +

labs(y="log2 expression", x = "sample",

title="Log2 Counts per Million (CPM)",

subtitle="filtered, TMM normalized",

caption=paste0("produced on ", Sys.time())) +

theme_bw()

plot_grid(p1, p2, p3, labels = c('A', 'B', 'C'), label_size = 12)

**Step02: PCA Plot**

# Libraries required----

library(tidyverse)

library(DT)

library(gt)

library(plotly)

# Identify variables of interest in study design file ----

targets <- read_tsv("studydesign.txt")

group <- targets$Group

group <- factor(group)

# Prepare your data -------

log2.cpm.filtered.norm.df

# Hierarchical clustering ----

distance <- dist(t(log2.cpm.filtered.norm), method = "maximum") #other distance methods are "euclidean", maximum", "manhattan", "canberra", "binary" or "minkowski"

clusters <- hclust(distance, method = "average") #other agglomeration methods are "ward.D", "ward.D2", "single", "complete", "average", "mcquitty", "median", or "centroid"

plot(clusters, labels=sampleLabels)

# Principal component analysis (PCA) ------

pca.res <- prcomp(t(log2.cpm.filtered.norm), scale.=F, retx=T)

ls(pca.res)

summary(pca.res)

pca.res$rotation

pca.res$x

screeplot(pca.res)

pc.var<-pca.res$sdev^2

pc.per<-round(pc.var/sum(pc.var)*100, 1)

pc.per

plot(pc.per)

# Visualize your PCA result ---

pca.res.df <- as_tibble(pca.res$x)

pca.plot <- ggplot(pca.res.df) +

aes(x=PC1, y=PC2, label=sampleLabels, color=group) +

geom_point(size=7) +

#geom_label() +

#stat_ellipse() +

xlab(paste0("PC1 (",pc.per[1],"%",")")) +

ylab(paste0("PC2 (",pc.per[2],"%",")")) +

labs(title="PCA plot",

caption=paste0("produced on ", Sys.time())) +

coord_fixed() +

theme_bw()

plot(pca.plot)

#mydata----

mydata.df <- log2.cpm.filtered.norm.df %>%

mutate(control.AVG = (A2 + A5)/2,

tub_ppx.AVG = (P3 + P4)/2,

logFC.tub_ppx_WT = (tub_ppx.AVG - control.AVG))

mydata.df

write.csv(mydata.df, file = "Log2.Filtered.Normalized.Genes.List.tsv")

**Step03: GSEA**

#libraries required----

library(clusterProfiler)

library(msigdbr)

library(gt)

library(DT)

library(plotly)

library(enrichplot)

library(org.Dm.eg.db)

# Perform GSEA using clusterProfiler ----

dm_gsea <- msigdbr(species = "Drosophila melanogaster")

dm_gsea %>%

dplyr::distinct(gs_cat, gs_subcat) %>%

dplyr::arrange(gs_cat, gs_subcat)

dm_gsea_c2 <- msigdbr(species = "Drosophila melanogaster",

category = "C2") %>%

dplyr::select(gs_name, gene_symbol)

mydata.df.polyP.WT <- dplyr::select(mydata.df, geneID, logFC.tub_ppx_WT)

mydata.gsea.polyP.WT <- mydata.df.polyP.WT$logFC.tub_ppx_WT

names(mydata.gsea.polyP.WT) <- as.character(mydata.df.polyP.WT$geneID)

mydata.gsea.polyP.WT <- sort(mydata.gsea.polyP.WT, decreasing = TRUE)

myGSEA.res.polyP.WT <- GSEA(mydata.gsea.polyP.WT, TERM2GENE=dm_gsea_c2, verbose=FALSE)

myGSEA.res.polyP.WT.df <- as_tibble(myGSEA.res.polyP.WT@result)

myGSEA.res.polyP.WT.df <- myGSEA.res.polyP.WT.df %>%

mutate(phenotype = case_when(

NES > 0 ~ "Upregulated",

NES < 0 ~ "Downregulated"))

myGSEA.plot.WT.polyP <- ggplot(myGSEA.res.polyP.WT.df[1:10,], aes(x=phenotype, y=ID)) +

geom_point(aes(size=setSize, color = NES, alpha=-log10(p.adjust))) +

scale_color_gradient(low="blue", high="red") + coord_fixed() +

theme_bw()

plot(myGSEA.plot.WT.polyP)

datatable(myGSEA.res.polyP.WT.df,

extensions = c('KeyTable', "FixedHeader"),

caption = 'Pathways enriched in TubPPX compare to WT-Control',

options = list(keys = TRUE, searchHighlight = TRUE, pageLength = 10, lengthMenu = c("10", "25", "50", "100"))) %>%

formatRound(columns=c(2:10), digits=2)

# create enrichment plots using the enrichplot package

gseaplot2(myGSEA.res.polyP.WT,

geneSetID = c(2, 32, 11), #can choose multiple signatures to overlay in this plot

pvalue_table = FALSE, #can set this to FALSE for a cleaner plot

title = myGSEA.res.polyP.WT$Description[105]) #can also turn off this title

gseaplot2(myGSEA.res.polyP.WT,

geneSetID = c(34, 40), #can choose multiple signatures to overlay in this plot

pvalue_table = FALSE, #can set this to FALSE for a cleaner plot

title = myGSEA.res.polyP.WT$Description[105]) #can also turn off this title

# Perform gseGO using clusterprofiler----

myGSEA.GO.polyP.WT.BP <- gseGO(geneList=mydata.gsea.polyP.WT,

ont ="BP",

keyType = "SYMBOL",

#nPerm = 10000,

pvalueCutoff = 0.05,

verbose = TRUE,

OrgDb = "org.Dm.eg.db",

pAdjustMethod = "BH")

myGSEA.GO.polyP.WT.BP.df <- as_tibble(myGSEA.GO.polyP.WT.BP@result)

write.csv(myGSEA.GO.polyP.WT.BP.df, file = "GSEA.GO.BP.tsv")

require(DOSE)

dotplot(myGSEA.GO.polyP.WT.BP, showCategory=5, split=".sign", font.size = 20) + facet_grid(.~.sign)

datatable(myGSEA.GO.polyP.WT.BP.df,

extensions = c('KeyTable', "FixedHeader"),

caption = 'Pathways enriched in TubPPX compare to WT-Control',

options = list(keys = TRUE, searchHighlight = TRUE, pageLength = 10, lengthMenu = c("10", "25", "50", "100"))) %>%

formatRound(columns=c(2:10), digits=2)

gseaplot2(myGSEA.GO.polyP.WT.BP,

geneSetID = c(5,92),

pvalue_table = FALSE,

base_size = 20)

myGSEA.GO.polyP.WT.MF <- gseGO(geneList=mydata.gsea.polyP.WT,

ont ="MF",

keyType = "SYMBOL",

#nPerm = 10000,

pvalueCutoff = 0.05,

verbose = TRUE,

OrgDb = "org.Dm.eg.db",

pAdjustMethod = "BH")

myGSEA.GO.polyP.WT.MF.df <- as_tibble(myGSEA.GO.polyP.WT.MF@result)

write.csv(myGSEA.GO.polyP.WT.MF.df, file = "GSEA.GO.MF.tsv")

require(DOSE)

dotplot(myGSEA.GO.polyP.WT.MF, showCategory=5, split=".sign", font.size = 20) + facet_grid(.~.sign)

myGSEA.GO.polyP.WT.CC <- gseGO(geneList=mydata.gsea.polyP.WT,

ont ="CC",

keyType = "SYMBOL",

#nPerm = 10000,

pvalueCutoff = 0.05,

verbose = TRUE,

OrgDb = "org.Dm.eg.db",

pAdjustMethod = "BH")

myGSEA.GO.polyP.WT.CC.df <- as_tibble(myGSEA.GO.polyP.WT.CC@result)

write.csv(myGSEA.GO.polyP.WT.CC.df, file = "GSEA.GO.CC.tsv")

require(DOSE)

dotplot(myGSEA.GO.polyP.WT.CC, showCategory=5, split=".sign", font.size = 20) + facet_grid(.~.sign)

**Step04: Heatmap of GSEA**

#libraries----

library(tidyverse)

library(dplyr)

library(gplots)

#Heatmap of GSEA Cytoplasmic Translation----

myGSEA.GO.polyP.WT.BP.df

GSEACytotranslation <- myGSEA.GO.polyP.WT.BP.df[20,11]

write.table(myGSEA.GO.polyP.WT.BP.df[20,11] ,file = "GSEA_CytoTranslation.tsv", sep =" ", row.names = FALSE)

GSEA_CytoTranslation <- read_tsv("GSEA_CytoTranslation.txt")

GSEA_CytoTranslation.df <- as_tibble(GSEA_CytoTranslation)

GSEA_CytoTranslation.Exp.df <- (semi_join(mydata.df, GSEA_CytoTranslation.df, by="geneID"))

GSEA_CytoTranslation.Exp.df <- GSEA_CytoTranslation.Exp.df[,1:5]

colnames(GSEA_CytoTranslation.Exp.df) <- c("geneID", "Control","Control", "Cyto_FLYX", "Cyto_FLYX")

GSEA_CytoTranslation.Exp <- as.matrix(sapply(GSEA_CytoTranslation.Exp.df[,2:5], as.numeric))

rownames1 <- GSEA_CytoTranslation.Exp.df[,1]

rownames1 <- as.matrix(sapply(rownames1, as.character))

rownames(GSEA_CytoTranslation.Exp) <- rownames1

clustRows.GSEA_CytoTranslation.Exp <- hclust(as.dist(1-cor(t(GSEA_CytoTranslation.Exp), method =

"pearson")), method ="complete")

clustColumns.GSEA_CytoTranslation.Exp <- hclust(as.dist(1-cor(GSEA_CytoTranslation.Exp, method="spearman")),

method="complete")

module.assign <- cutree(clustRows.GSEA_CytoTranslation.Exp, k=2)

module.color <- rainbow(length(unique(module.assign)), start=0.1, end=0.9)

module.color <- module.color[as.vector(module.assign)]

heatmap.2(GSEA_CytoTranslation.Exp,

Rowv=as.dendrogram(clustRows.GSEA_CytoTranslation.Exp),

Colv=NA,

RowSideColors=module.color,

col=rev(myheatcolors2), scale='row', labRow=clustRows.GSEA_CytoTranslation.Exp$labels,

density.info="none", trace="none",

cexRow=1, cexCol=1, margins=c(10,20),

keysize = 1,

lhei = c(1,4))

#Heatmap of GSEA Mitochondrial Translation----

myGSEA.GO.polyP.WT.BP.df

GSEAMitotranslation <- myGSEA.GO.polyP.WT.BP.df[36,11]

write.table(myGSEA.GO.polyP.WT.BP.df[36,11] ,file = "GSEA_MitoTranslation.tsv", sep =" ", row.names = FALSE)

GSEA_MitoTranslation <- read_tsv("GSEA_MitoTranslation.txt")

GSEA_MitoTranslation.df <- as_tibble(GSEA_MitoTranslation)

GSEA_MitoTranslation.Exp.df <- (semi_join(mydata.df, GSEA_MitoTranslation.df, by="geneID"))

GSEA_MitoTranslation.Exp.df <- GSEA_MitoTranslation.Exp.df[,1:5]

colnames(GSEA_MitoTranslation.Exp.df) <- c("geneID", "Control","Control", "Cyto_FLYX", "Cyto_FLYX")

GSEA_MitoTranslation.Exp <- as.matrix(sapply(GSEA_MitoTranslation.Exp.df[,2:5], as.numeric))

rownames1 <- GSEA_MitoTranslation.Exp.df[,1]

rownames1 <- as.matrix(sapply(rownames1, as.character))

rownames(GSEA_MitoTranslation.Exp) <- rownames1

clustRows.GSEA_MitoTranslation.Exp <- hclust(as.dist(1-cor(t(GSEA_MitoTranslation.Exp), method =

"pearson")), method ="complete")

clustColumns.GSEA_MitoTranslation.Exp <- hclust(as.dist(1-cor(GSEA_MitoTranslation.Exp, method="spearman")),

method="complete")

module.assign <- cutree(clustRows.GSEA_MitoTranslation.Exp, k=2)

module.color <- rainbow(length(unique(module.assign)), start=0.1, end=0.9)

module.color <- module.color[as.vector(module.assign)]

heatmap.2(GSEA_MitoTranslation.Exp,

Rowv=as.dendrogram(clustRows.GSEA_MitoTranslation.Exp),

Colv=NA,

RowSideColors=module.color,

col=rev(myheatcolors2), scale='row', labRow=clustRows.GSEA_MitoTranslation.Exp$labels,

density.info="none", trace="none",

cexRow=1, cexCol=1, margins=c(10,20),

keysize = 1,

lhei = c(1,4))

#Heatmap of GSEA Ribosome biogenesis----

myGSEA.GO.polyP.WT.BP.df

GSEARibosomeBiogenesis <- myGSEA.GO.polyP.WT.BP.df[92,11]

write.table(myGSEA.GO.polyP.WT.BP.df[92,11] ,file = "GSEA_RibosomeBiogenesis.tsv", sep =" ", row.names = FALSE)

GSEA_RibosomeBiogenesis <- read_tsv("GSEA_RibosomeBiogenesis.txt")

GSEA_RibosomeBiogenesis.df <- as_tibble(GSEA_RibosomeBiogenesis)

GSEA_RibosomeBiogenesis.Exp.df <- (semi_join(mydata.df, GSEA_RibosomeBiogenesis.df, by="geneID"))

GSEA_RibosomeBiogenesis.Exp.df <- GSEA_RibosomeBiogenesis.Exp.df[,1:5]

colnames(GSEA_RibosomeBiogenesis.Exp.df) <- c("geneID", "Control","Control", "Cyto_FLYX", "Cyto_FLYX")

GSEA_RibosomeBiogenesis.Exp <- as.matrix(sapply(GSEA_RibosomeBiogenesis.Exp.df[,2:5], as.numeric))

rownames1 <- GSEA_RibosomeBiogenesis.Exp.df[,1]

rownames1 <- as.matrix(sapply(rownames1, as.character))

rownames(GSEA_RibosomeBiogenesis.Exp) <- rownames1

clustRows.GSEA_RibosomeBiogenesis.Exp <- hclust(as.dist(1-cor(t(GSEA_RibosomeBiogenesis.Exp), method =

"pearson")), method ="complete")

clustColumns.GSEA_RibosomeBiogenesis.Exp <- hclust(as.dist(1-cor(GSEA_RibosomeBiogenesis.Exp, method="spearman")),

method="complete")

module.assign <- cutree(clustRows.GSEA_RibosomeBiogenesis.Exp, k=2)

module.color <- rainbow(length(unique(module.assign)), start=0.1, end=0.9)

module.color <- module.color[as.vector(module.assign)]

heatmap.2(GSEA_RibosomeBiogenesis.Exp,

Rowv=as.dendrogram(clustRows.GSEA_RibosomeBiogenesis.Exp),

Colv=NA,

RowSideColors=module.color,

col=rev(myheatcolors2), scale='row', labRow=clustRows.GSEA_RibosomeBiogenesis.Exp$labels,

density.info="none", trace="none",

cexRow=1, cexCol=1, margins=c(10,20),

keysize = 1,

lhei = c(1,4))
